# Acute Cardiac Tamponade During Multiple Electrical Pre‐Mappings for Atrial Leadless Pacemaker Implantation

**DOI:** 10.1002/joa3.70202

**Published:** 2025-10-07

**Authors:** Ryuki Chatani, Akina Takauchi, Mitsuru Yoshino, Hiroshi Tasaka, Kazushige Kadota

**Affiliations:** ^1^ Department of Cardiovascular Medicine Kurashiki Central Hospital Kurashiki Japan

**Keywords:** Aveir‐AR, cardiac tamponade, helix‐based active‐fixation leadless pacemaker, leadless pacemaker

## Abstract

Although cardiac tamponade during atrial leadless pacemaker implantation (ALPI) is rare, we encountered such an incident in a female patient with a lower body mass index during multiple pre‐mapping attempts. During ALPI, operators should aim for successful single‐attempt deployment using electrical pre‐mapping as few times as possible to prevent cardiac tamponade.

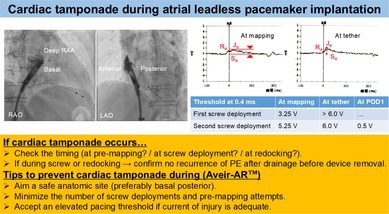

## 1 Introduction

In leadless pacemaker implantation (LPI), the occurrence of acute cardiac tamponade must be strictly avoided. The introduction of an atrial LPI (ALPI) system (Aveir‐AR; Abbott, USA) is anticipated to increase the use of LPI systems, including both single‐chamber (AAI) and dual‐chamber DDD systems. Active‐fixation LPI systems are expected to reduce the risk of cardiac tamponade by minimizing the number of deployments through multiple contact mappings of electric parameters prior to fixation. Herein, we present the case of a patient with sick sinus syndrome (SSS) who underwent ALPI with a single deployment, after which repeated multiple contact mapping attempts resulted in the development of acute cardiac tamponade.

## 2 Case Description

The patient was a 58‐year‐old woman with a history of epilepsy, for which she had been prescribed Tegretol. She was hospitalized for syncope due to SSS type II without evidence of type I. Her body weight and body mass index were 47.5 kg and 19.9 kg/m^2^, respectively. Because she had only a transient long pause (max R‐R interval, 14.7 s) and the expected pacing ratio was very low, ALPI with Aveir‐AR in AAI mode was planned.

**FIGURE 1:**
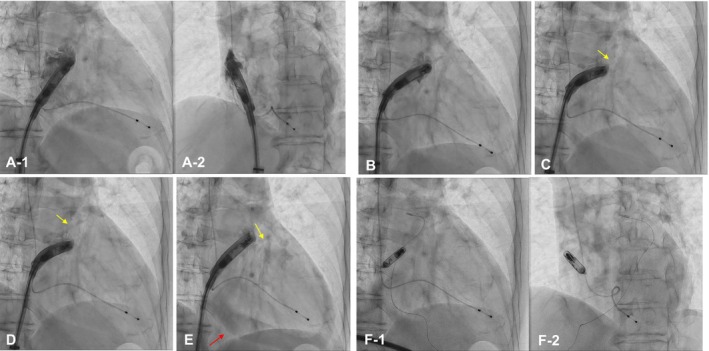
Fluoroscopic image of atrial leadless pacemaker implantation (ALPI) in right anterior oblique (RAO) view (A‐1) and left anterior oblique (LAO) view (A‐2) at the first attempt. (B) Fluoroscopic image showing the fifth mapping position in the RAO view. (C) Fluoroscopic image showing the sixth mapping position in the RAO view with right atrial appendage (RAA) extravasation (yellow arrow). (D) Fluoroscopic image showing the seventh mapping position in the RAO view with RAA extravasation (yellow arrow). (E) Fluoroscopic image showing the eleventh mapping position in the RAO view with RAA extravasation (yellow arrow) and pericardial effusion (red arrow). (F) Fluoroscopic image of ALPI in the RAO view (F‐1) and LAO view (F‐2) after device release.

**FIGURE 2:**
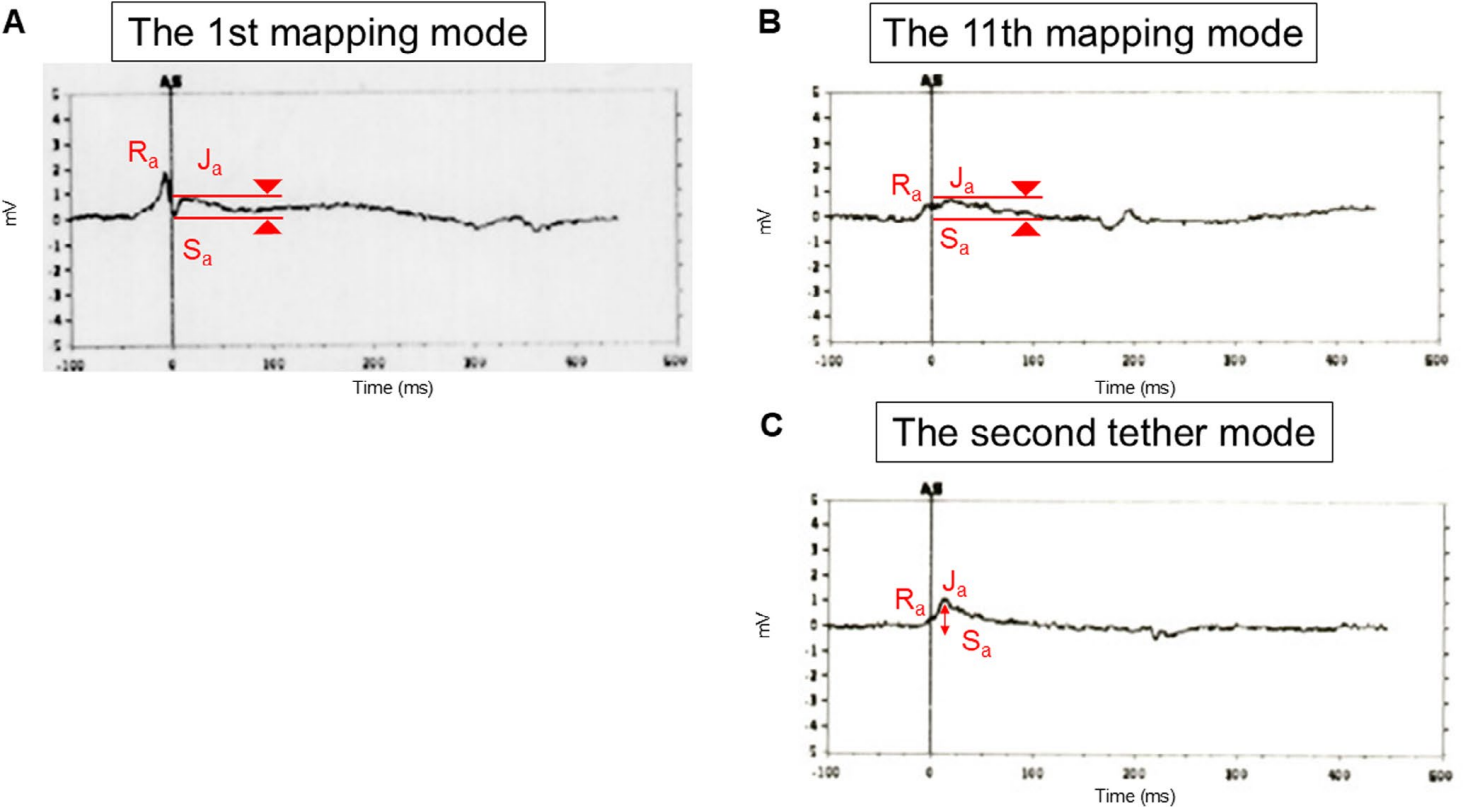
Commanded intracardiac electrograms from the Aveir‐AR during ALPI. (A) At the first mapping. (B) At the 11th mapping. (C) At the second tether mode.

At the first attempt, Aveir‐AR was deployed on the posterior base of the right atrial appendage (RAA) (Figure 1A; Video 1). The commanded intracardiac electrograms during the first mapping indicated advancement into responsive healthy tissue (Figure 2A). However, the pacing capture threshold and sensed amplitude at tether mode were unacceptable (4.0 V at 1.5 ms and < 0.25 mV), and evaluation of the commanded intracardiac electrogram was hindered by the extremely low sensed amplitude. Therefore, we subsequently retrieved the device for repositioning 8 min after the first fixation. After retrieval of the device from the first attempt due to unacceptable pacing capture threshold or sensed amplitude, an additional 10 rounds of contact pre‐mapping were performed (Table 1). The fifth, sixth, seventh, and eleventh RAA angiograms were shown in Figure 1B (Video 2), Figure 1C (Video 3), Figure 1D (Video 4), and Figure 1E (Video 5), respectively. At the 11th round of contact pre‐mapping, the sensed amplitude and pacing capture threshold were acceptable. The commanded intracardiac electrogram during this mapping is shown in Figure 2B. The Aveir‐AR was successfully placed on the more posterior and mid‐portion of the RAA (Figure 1F). The commanded intracardiac electrogram at tether mode was characterized by a positive shift in the Sa‐Ja segment (Figure 2C).

**VIDEO 1:** Right atrial appendage (RAA) angiogram showing the first mapping position and attempted position. Video content can be viewed at https://onlinelibrary.wiley.com/doi/10.1002/joa3.70202.

**TABLE 1:** Electrical mapping data.

Electrical mapping	Threshold	Sense (mV)	Impedance (ohms)
Initial mapping at IVC	—	—	310
The 1st mapping	3.25 V at 0.4 ms	1.5	330
The 1st tether mode	4.0 V at 1.5 ms	< 0.25	380
The 2nd mapping	—	< 0.5	330
The 3rd mapping	4.0 V at 1.5 ms	< 0.25	320
The 4th mapping	> 6.0 V at 1.5 ms	0.9	320
The 5th mapping	> 6.0 V at 1.5 ms	—	—
The 6th mapping	5.25 V at 1.5 ms	0.6	300
The 7th mapping	—	< 0.5	270
The 8th mapping	4.75 V at 1.5 ms > 6.0 V at 0.4 ms	0.6	510
The 9th mapping	—	< 0.5	—
The 10th mapping	—	< 0.5	—
The 11th mapping	3.25 V at 1.5 ms 5.25 V at 0.4 ms	0.6	300
The 2nd tether mode	3.25 V at 1.5 ms 6.0 V at 0.4 ms	0.7	330

Abbreviation: IVC, inferior vena cava.

**VIDEO 2:** RAA angiogram showing the fifth mapping position. Video content can be viewed at https://onlinelibrary.wiley.com/doi/10.1002/joa3.70202.

**VIDEO 3:** RAA angiogram showing the sixth mapping position. Video content can be viewed at https://onlinelibrary.wiley.com/doi/10.1002/joa3.70202.

**VIDEO 4:** RAA angiogram showing the seventh mapping position. Video content can be viewed at https://onlinelibrary.wiley.com/doi/10.1002/joa3.70202.

**VIDEO 5:** RAA angiogram showing the 11th mapping position and second attempted position. Video content can be viewed at https://onlinelibrary.wiley.com/doi/10.1002/joa3.70202.

The pacing threshold was 3.5 V at 1.5 ms, the P‐wave amplitude was 0.7 mV, and the impedance was 330 Ω at tether mode (Figure 1E; red arrow). However, pericardial effusion was observed on fluoroscopy, and cardiac tamponade was diagnosed by echocardiography at that time. We immediately reversed the heparin and performed percutaneous pericardial drainage, removing 400 mL of bloody pericardial effusion. Retrospectively, RAA perforation was suspected to have occurred between the fifth (Figure 1B; Video 2) and sixth (Figure 1C; Video 3) RAA angiograms, with the fifth RAA angiogram (Video 2) showing contrast injection and the helix of the device unintentionally exposed. At the time of suspected perforation, commanded intracardiac electrogram data were not available; however, contemporaneous notes indicated no significant changes, including ST‐segment depression. Pericardial effusion gradually increased over 27 min, leading to cardiac tamponade. As the pericardial effusion did not recur within 5 min after drainage and the final deployment was not considered to be related to the effusion, a tension test was performed, and the device was released (pacing threshold 3.25 V at 1.5 ms, P‐wave amplitude 0.5 mV, impedance 330 Ω). The total procedure time and contrast volume were 192 min and 106 mL, respectively. On the following day, the Aveir‐AR electrical parameters had improved, with a pacing threshold of 0.5 V at 0.4 ms, a P‐wave amplitude of 0.6 mV, and an impedance of 480 Ω. Drain output was 20 mL over 12 h, and the drain was removed. Pericardial effusion did not recur, and she was discharged 5 days after drain removal. One month after ALPI, the Aveir‐AR electrical parameters were within normal limits (constant voltage 3.0 V, battery longevity ≥ 14.5 years, pacing threshold 0.5 V at 0.4 ms, P‐wave 0.8 mV, impedance 390 Ω), and the atrial pacing rate was 1% (AAI 50 mode). There was no recurrence of pericardial effusion.

## 3 Discussion

In LPI, both an increased number of deployments and the use of a validated risk score for predicting pericardial effusion have been reported [1]. This patient was a female with a lower body mass index and was considered high risk. High‐risk cases are more likely to develop pericardial effusion because of the increased number of deployments. A recent study reported that the Aveir‐AR was successfully implanted on a single attempt in 78% of cases, and that 60% of cases were successfully implanted without requiring ≥ 2 mapping attempts [2]. Therefore, it would have been preferable to aim for a successful single‐attempt deployment with as few mappings as possible. An important consideration is whether an elevated pacing threshold at the first attempt during ALPI should be accepted or whether repositioning should be performed, as in this case. A recent study reported significant improvements from implant to 1 month in both pacing capture threshold (2.4 ± 1.5–0.8 ± 0.8 V at 0.4 ms; *p* < 0.001) and sensed amplitude (1.8 ± 1.3–3.4 ± 1.9 mV; *p* < 0.001) [3]. The improvement in pacing threshold may be attributed to the resolution of acute tissue injury and inflammation resulting from the Aveir‐AR inner and outer helix fixation, whereas the improvement in sensed amplitude may be related to fibrotic tissue ingrowth and potential encapsulation of the distal end of the Aveir‐AR.

In addition, a recent study reported that a high pacing threshold (≥ 3.0 V at 0.4 ms) during ALPI decreased to 1.1 ± 1.0 V at 0.4 ms within 24 h and remained stable thereafter [4]. The presence of an adequate current of injury (COI) and an elevated pacing threshold during ALPI should be accepted, and unnecessary repositioning should be avoided. It is important to note that in this case, the commanded intracardiac electrogram demonstrated a poor tissue response due to repeated mapping. We could have waited longer after the first deployment to observe potential improvements in sensed amplitude, COI, and pacing threshold before releasing the device. In addition, in this case, the fifth RAA angiogram (Video 2) showed the helix of the device unintentionally exposed, which may have contributed to damage of the RAA. Operators should deliver the device and perform RAA angiography while the sleeve is still covering and protecting the distal end of the LPI device.

To prevent cardiac tamponade, we should identify a safe anatomic location (preferably closer to the basal posterior site) using contrast imaging and/or intracardiac echocardiography, confirm an adequate electrical COI, minimize the number of repositionings and electrical pre‐mapping attempts, and consider converting transvenous pacemaker implantation in challenging cases.

In addition, when cardiac injury occurs, the timing should be carefully reviewed retrospectively to determine whether it was due to the screw deployment. If screw deployment is not associated and hemodynamic stability is maintained without recurrence of pericardial effusion, the device can be released. Importantly, if cardiac tamponade occurs during LPI, the device should not be hastily removed [5].

## Ethics Statement

Approval was obtained from the local ethics committee.

## Consent

Written informed consent was obtained from the patient for publication of this case report.

## Conflicts of Interest

The authors declare no conflicts of interest.
